# Bridging Biological Multiscale Structure and Biomimetic Ceramic Construction

**DOI:** 10.34133/research.0588

**Published:** 2025-02-10

**Authors:** Jingjiang Wei, Tianyu Yuan, Hang Ping, Fei Pan, Zhengyi Fu

**Affiliations:** ^1^ Institute for Advanced Study, Chengdu University, Chengdu 610106, P. R. China.; ^2^ Hubei Longzhong Laboratory, Wuhan University of Technology Xiangyang Demonstration Zone, Xiangyang 441000, P. R. China.; ^3^ State Key Laboratory of Advanced Technology for Materials Synthesis and Processing, Wuhan University of Technology, Wuhan 430070, P. R. China.; ^4^ Department of Chemistry, University of Basel, Basel 4058, Switzerland.

## Abstract

The brittleness of traditional ceramics severely limits their application progress in engineering. The multiscale structural design of organisms can solve this problem, but it still lacks sufficient research and attention. The underlined main feature is the multiscale hierarchical structures composed of basic nano–microstructure units arranged in order, which is currently impossible to achieve through artificial synthesis driven by high temperatures. This perspective aims to bridge the gap between biostructural materials and biomimetic ceramics, highlighting the relationship between bioinspired structures and interfacial interaction of structure densification in biomimetic ceramics. Therefore, we could accomplish densification and ceramic development at room temperature, consequently correlating the structure, properties, and functions of materials and accelerating the development of the next generation of advanced functional ceramics.

## Introduction

Ceramics are essential for people’s lives, high-tech industry, and modern national defense, which additionally have remarkable characteristics, such as a high melting point, distinctive hardness, excellent wear resistance, oxidation resistance, low thermal conductivity, and electrical conductivity [1]. However, the preparation method of ceramic materials usually involves sintering at a temperature over 1,000 °C, which not only consumes substantial energy but also causes severe environmental pollution. Therefore, it is very desirable to prepare functional ceramics at room temperature or low temperature [2]. Some biostructural materials in nature combine inorganic hard components and organic soft components and are organized from the nanoscale to the macroscale under environmental conditions to realize various complex biofunctions and biostructures [3,4]. For example, the multiscale hierarchical structure of nacre and enamel has excellent mechanical strength and toughness [3–5]. Similarly, the Bouligand structure found in fish scales, lobster claws, and insect shells enables them to display outstanding mechanical properties and unique structural colors [3–5]. Therefore, inspired by the multiscale structure of natural bioceramics, preparing biomimetic ceramic materials at room temperature is an innovative technology that subverts the need for high-temperature sintering of ceramics and utilizes the advantages of biomaterials. Thus, we can develop novel functional ceramic materials toward higher standards.

## Formation Mechanism of Multiscale Structural Bioceramics

Traditional ceramics are polycrystalline materials formed by high-temperature natural or synthetic sintering through atomic diffusion driven by high temperature. However, the pores and impurities at the grain boundaries in ceramics induce cracks, which cause ceramic interior fragility to external force. Compared with ceramics composed of pure inorganic minerals, organisms sacrifice a part of inorganic phases and introduce a small amount of organic matter to form multiscale-ordered bioceramics. Moreover, their mechanical properties are not observably reduced but manifest greatly improved toughness. The representative bioceramic is nacre with bright color and excellent material properties, which is mainly composed of inorganic minerals (95% aragonite by volume) and a small amount of organic matrix (1% to 5% β-chitin and fibroin by volume) [4,5]. Due to the multiscale brick-and-mortar structure of nacre (Figure A) [4,6], it has an extraordinary damage tolerance. At the molecular level, β-chitin/fibroin combines free ions to form stable organic–inorganic composite precursors, forming mesoscopic aragonite sheets at the nanoscale. The organic matter between nanoparticles and the interface between aragonite nanosheets confer resistance toward large pressure or strain under high tension. Furthermore, aragonite microplates filled and connected by interlayer organic layers can be configured with Thiessen polygonal patterns, completely interconnected in chitin’s continuous organic skeleton, thus forming a macroscopic nacre. Therefore, the strength and toughness of nacre are attributed to several key factors in its formation process, including an ordered microstructure, interface connection, and the regulation of proteins and ions, which are of great importance for the preparation of ceramics at room temperature.

**Figure.**
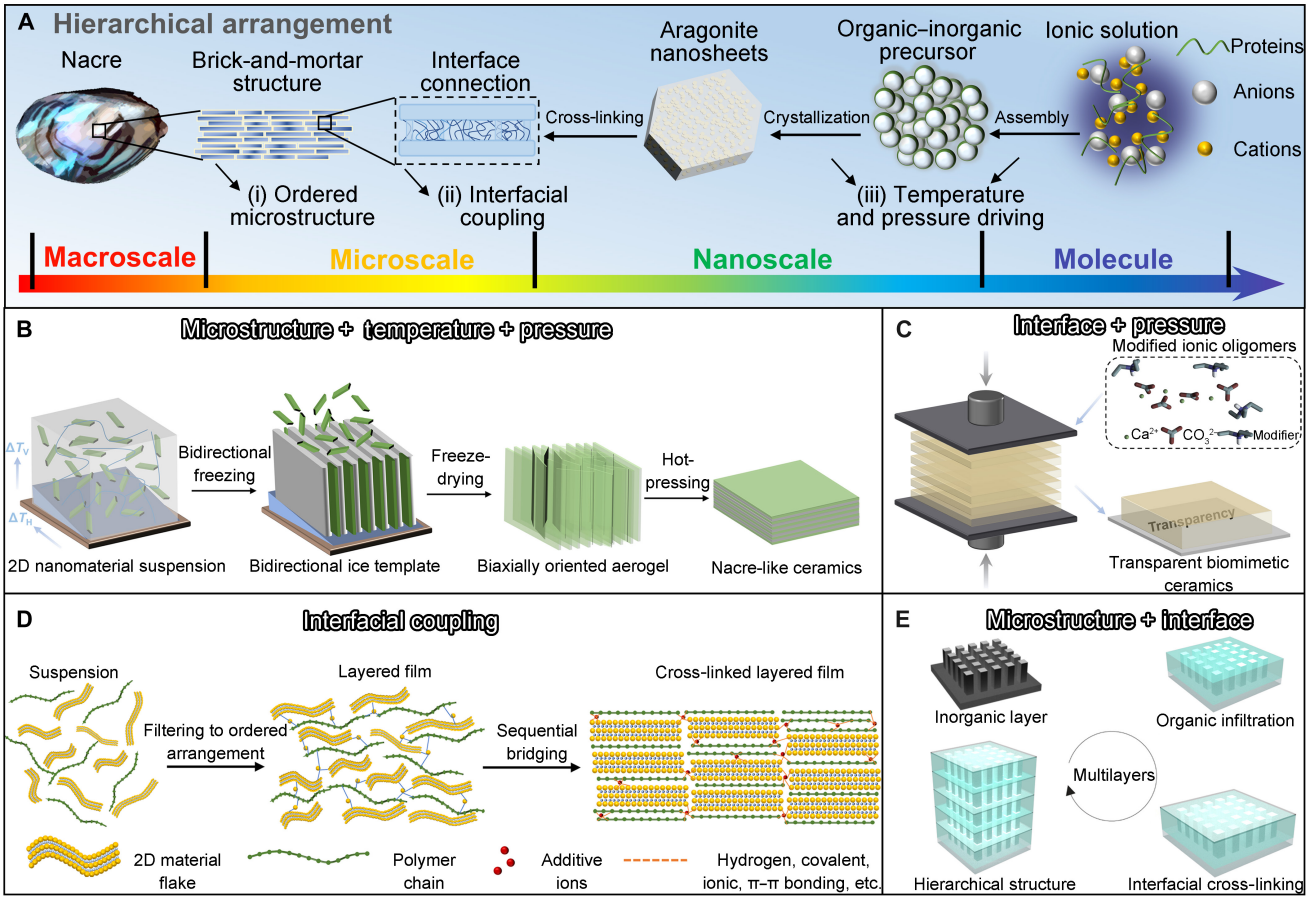
Technical conceptual illustration of biomimetic ceramic preparation. (A) Multiscale hierarchical structure of natural nacre. (B) Nacre-like ceramics prepared by combining freeze-casting and hot-pressing techniques. (C) Pressure-driven organic–inorganic cross-linking ionic oligomers for transparent biomimetic ceramics. (D) Continuous bridging technology for dense layered films. (E) Layer-by-layer technology for microadditive manufacturing of hierarchical structural materials. 2D, 2-dimensional.

## Hierarchical Structure Design Strategy of Biomimetic Ceramics

Conventional preparation techniques, such as self-assembly, the layer-by-layer technique, and 3-dimensional printing, can only simply simulate the single characteristics of biological structural materials. However, the optimal synergistic performance of natural bioceramics is not just a simple material accumulation. Therefore, to design biomimetic ceramics with properties equivalent to or even better than those of natural nacre, it is necessary to systematically combine the key factors of nacre and the ceramic formation process, including (a) formation of an ordered microstructure, (b) interfacial coupling between layers, and (c) appropriate driving forces, such as temperature and pressure.

The combination of freeze-casting and hot-pressing technology is often used to prepare bulk materials. Hence, 2-dimensional nanomaterials, illustrated in Figure B, can sophisticatedly form nacre-like layered microstructures through directional freeze-induced assembly. Herein, the control of temperature and pressure can finally drive the formation of dense biomimetic nacre [7,8]. The combination of microstructure, temperature, and pressure realized the rapid preparation of nacre-like ceramics. However, accomplishing the densification of ceramics at room temperature is still challenging. Tang et al. [9–11] solved this challenge by designing a series of oligomers of mineral precursors. Moreover, through interfacial cross-linking (organic–inorganic oligomer precursor) and pressure, biomimetic ceramics with transparency and even elasticity were rapidly densified at room temperature (Figure C). Generally, the formation process of biostructural materials is often at ambient pressure despite being at room temperature. Inspired by various interfacial coupling methods of nacre (mineral bridge, inorganic mineral protrusion, organic binder, etc.), Cheng et al. [12–14] utilized hydrogen bonds, covalent bonds, ionic bonds, and π–π bonds to bridge the interface between organic and inorganic phases, which markedly reduced the porosity and realized the rapid densification of nacre-like materials at room temperature and ambient pressure (Figure D). The growth process of natural organisms is a typical additive manufacturing process. Therefore, bioinspired materials with adjustable functionality can be realized by microadditive manufacturing. Biomimetic hierarchical ceramics with a controllable thickness can thus be efficiently fabricated by adjusting multiple types of ordered microstructures and interface coupling (Figure E) [15–18]. Therefore, the synergistic combination of factors, such as microstructure, interface, temperature, and pressure, can boost the development of biomimetic ceramics with a function similar to or even better than that of the target natural analogs.

## Summary and Prospect

The existing preparation technology of biomimetic ceramics remarkably improves the toughness of materials and partially solves the problem of structure–function integration. However, they still face challenges such as low extreme mechanical strength, difficulty in large-scale production, and insufficient environmental tolerance. The established knowledge will guide us to develop technologies related to the design of advanced ceramics at low or room temperature. Future innovation should fully understand the multiscale structure of natural biomaterials and use the synergistic effect of microstructure, interface, temperature, and pressure to construct a synthetic hierarchical structure through strict regulation of morphology, structure, function, appearance, and mechanics to optimize the performance of various applications.

Biomimetic ceramics will be widely used in many fields in the future. Especially in the fields of aerospace and precision instruments, biomimetic ceramics show excellent damping performance and good fracture toughness, which are suitable for bearing and vibration reduction. In the medical field, biomimetic ceramics have excellent biocompatibility and chemical stability and can be applied to the repair and replacement of teeth, bones, and joints. In addition, biomimetic ceramics will also play a vital role in the field of intelligent building materials because of their light weight, high strength, and environmental protection.
